# Parental Happiness Socialization and Youth Adjustment in Italy and Azerbaijan in the COVID-19 Pandemic Era

**DOI:** 10.3390/ijerph20043604

**Published:** 2023-02-17

**Authors:** Carolina Lunetti, Laura Di Giunta, Giulia Gliozzo, Chiara Riccioni, Clementina Comitale, Emanuele Basili, Aysel Baxseliyeva, Alessia Teresa Virzì

**Affiliations:** 1 Department of Psychology, Sapienza University of Rome, Via dei Marsi, 78, 00175 Rome, Italy; 2 IRCCS Fondazione Santa Lucia, Via Ardeatina, 306/354, 00179 Roma, Italy

**Keywords:** parental emotion socialization, happiness, socio-emotional adjustment, academic performance, COVID-19, cultures, early adolescence

## Abstract

This study aims to cross-culturally identify the parental socialization strategies in response to a child’s happiness and their associations with youth academic and socio-emotional adjustment, controlling for the impact of the COVID-19 pandemic. Participants were a convenient sample of Italian (N = 606, 81.9% mothers) and Azerbaijanis (N = 227, 61.4% mothers) parents of youths (*M*_age_ = 12.89, *SD* = 4.06; 51% girls). Parents filled out an online survey to assess their socialization strategies in response to their children’s happiness, their children’s negative emotion regulation and dysregulation, academic performance, and prosocial behavior. Exploratory factorial analysis showed the presence of two factors that enclosed supportive and unsupportive parental socialization strategies. A multiple-group path analysis model showed that similarly across countries, supportive parental strategies were positively related to youths’ prosocial behavior and that unsupportive parental strategies were positively related to youths’ negative emotion dysregulation, and negatively related to youths’ academic performance and negative emotion regulation. Those results emerged controlling for parents’ and adolescents’ gender and age, parents’ educational level, social desirability, and Covid-related problems. This study advances cross-cultural knowledge about the impact of the strategies that parents use to socialize their children’s happiness in the unique context of the COVID-19 pandemic.

## 1. Introduction

According to the Ecological System Theory proposed by Bronfenbrenner [1], the individual’s development is determined by the interaction among multiple systems. Cross-sectional and longitudinal evidence supports the relevance of the family context (microsystem), and specifically of parents’ behaviors on youths’ long-term (mal)adjustment (e.g., [2]). Whereas examples of macrosystems are historical and societal changes affecting how parents socialize their children at cross-cultural levels.

Empirical evidence suggests that the challenges that families faced and continue to face in the 21st century have significantly increased compared to previous eras. For example, Brown and colleagues [3] highlighted how the high costs of childcare facilities, the inability of parents to reconcile the rhythms of family life with those of working life, the few rights of parents who work, the concern for the future of children, and the high cost of living and education, have intensified the dissatisfaction of parents in general, and have made parenting tasks more challenging. Those challenges are significantly intensified due to the recent event of the COVID-19 pandemic that has affected all people around the world. The COVID-19 pandemic and the restrictions imposed by national governments to prevent the contagion (e.g., lock-down, remote learning, smart-working) have drastically challenged the way by which parents have fulfilled their parental roles and their impact on youths’ development. Within this framework, recent studies are trying to show the impact that the COVID-19 pandemic has on the parent-child development relationship (e.g., [4]), and in particular, on the parent-child emotional development relationship (e.g., [5,6]). The results from these studies support that the COVID-19 pandemic has affected the impact of parental emotion socialization strategies on children’s adjustment in different ways; in particular during the pandemic, with schools closed, it was imposed on parents to become full-time childcare providers which, in addition to the few resources available, work and family management problems, and the impossibility of doing outdoor activities, may have negatively impact the way by which parents’ respond to their children’s emotional reactions and consequently, also the way by which children manage their emotional life and the challenged remote school and social contexts. The present study aims to innovatively contribute to providing empirical evidence on this nowadays scenario in multiple ways. First, it aims to study the impact of temporary parental emotion socialization on youth (mal)adjustment (academic performance, prosocial behaviors, emotion regulation, and dysregulation), controlling for the impact of the COVID-19 pandemic. Second, previous studies on parental emotion socialization mainly focused on youths’ negative emotions; however, we are not aware of previous studies focusing on parental socialization in response to youths’ positive emotions. Thus, the present study addresses this gap in the literature contributing to what parents do in response to their children’s positive emotional reactions. Third, recently, parental emotion socialization has begun to be examined with culturally diverse groups (e.g., [7,8]), showing that parental emotion socialization behaviors may not be similarly associated with adaptive child outcomes cross-culturally. Thus, this study expands the state of the art of parental emotion socialization and its relationships with youth adjustment (mostly capitalizing on North American samples, e.g., [9]) with a cross-cultural perspective. Indeed, the present study examines the association between parental emotion socialization and youth adjustment during COVID-19 times in two different cultural contexts that have been massively affected by the COVID-19 pandemic: Italy and Azerbaijan. The reasons for the choice to investigate these two cultural contexts is dependent on the facts that: (a) Italy was the first country in Europe to impose the national lockdown on 9 March 2020, and one of the European country most affected by the pandemic; (b) Azerbaijan, even with a little delay in the spread of infection, was massively affected by the pandemic; (c) to compare and possibly generalize the study’s findings in two completely different cultural contexts but representative of some important characteristics of European and Asian families.

### 1.1. Parental Emotion Socialization

Parental emotion socialization (PES) could be defined as the set of strategies that parents use to respond to their children’s emotional reactions [10]. In their heuristic model, Eisenberg et al. [10] explains that parental emotion-related socialization behaviors involve parents’ discussion about the emotion expressed by their children, and parents’ emotional reactions and expressions. Previous studies showed significant associations between the strategies that parents use to socialize their children’s emotions in affecting youths’ adjustment, emotional experience, and emotion regulation (e.g., [11]). A relevant study aimed at examining parental emotion socialization was conducted by Garside and Klimes-Dougan [12], using the Emotion as a Child Scale [13] on a sample of parents of adolescents. This study showed the presence of five supportive and unsupportive parental socialization strategies which include “Reward,” (supporting children to face negative emotions), “Override”, (distracting children from negative emotions), “Punish” (disapproving children’s expression of negative emotion), “Neglect” (avoiding children’s negative emotions), and “Magnify” (emphasizing children’s negative emotions).

Furthermore, a recent study conducted by Di Giunta and colleagues [6] on a sample of Italian parents of adolescents during the COVID-19 pandemic, supported the presence of supportive parental strategies (including the strategies originally conceptualized as “Reward” and “Override”) and unsupportive parental strategies (including the strategies originally conceptualized as “Punish”, “Neglect”, and “Magnify”) for the socialization of negative emotions (sadness, anger, and anxiety). This study also highlighted the positive association between supportive PES strategies and youth emotion regulation, as well as between unsupportive PES strategies and youth internalizing and externalizing problems.

The present study aims to examine the association of parental happiness socialization strategies with youth adjustment (academic achievement, prosocial behavior, and negative emotion regulation) and maladjustment (negative emotion dysregulation). Very few studies examined parental strategies in response to children’s positive emotions, such as happiness (hereafter, parental happiness socialization strategies). For example, relevant is the contribution of a recent study conducted by Lobo et al. [14], which showed that parents’ emotional coaching, which is a supportive strategy that refers to parents’ discussion about the consequences and causes of positive emotions, constitutes a protective factor to contrast the onset of internalizing problems during adolescence.

To guide hypotheses about the association between parental happiness socialization strategies and youth (mal)adjustment, we capitalized on previous studies on parental socialization strategies in response to youths’ negative emotions and youth adjustment. In addition, considering that “authoritative parents emphasize helping their children not only to understand and express their own emotions, but also to regulate the display of emotions” ([15], p. 646), we also capitalized on some previous studies examining the association between parenting and, specifically, academic achievement, prosocial behavior, and emotion (dys) regulation to infer some hypotheses about the corresponding associations between parental happiness socialization strategies as aforementioned youth-related outcomes.

### 1.2. Parents’ Behaviors and Youth Academic Achievement

Academic success is an important indicator of students’ positive adjustment and well-being across adolescence [16]. The impact of parenting style across all educational levels has been shown in several empirical contributions. Parents who are involved in their children’s scholastic matters and who believe that they can contribute to their children’s academic achievement can affect children’s intellectual and academic development [17,18]. Authoritative parenting, characterized by high levels of warmth and control in the parent-child relationship, was positively associated with high youth school performance in a Chinese American sample of parents [19], as well as in a Spanish one [20]. Similar results are supported by the study conducted by Abar and colleagues [21] which showed the impact of authoritative parenting in promoting African American high academic achievement, and by the study by Xiang and colleagues [22], which supported the direct and indirect effects of an authoritative parenting style in determining adolescents’ school adjustment. Relevant to this study can also be the work by Carmona-Halty et al. [23] highlighting the mediating role of positive parenting in the association between adolescents’ positive emotions and their school performance. Another relevant study conducted by Serna and Martinez [24] on a sample of Spanish adolescents supported that high parental involvement in their children’s life and growth positively affects adolescents’ school adjustment in terms of school integration and satisfaction.

To sum up, considering previous studies on the positive association between positive/authoritative parenting and youth school performance, it might be possible to hypothesize also a positive association between supportive PES and youth academic performance, as well as a negative association between unsupportive PES and youth academic performance.

Finally, few studies examined the role of culture in the association between parenting and school performance. For example, Lansford et al. [2] found that across nine countries, better school performance in late childhood was predicted by high parental warmth/affection and low psychological control [2]. Certainly, more studies are in need to explore, and eventually confirm, the direction of the aforementioned results from a cross-cultural perspective.

### 1.3. Parents’ Behaviors and Youth Prosocial Behavior

Prosocial behavior is generally defined as voluntary behavior aimed to help and share with others [25]. Many studies supported its impact on youths’ adjustment and positive development, e.g., [26,27]. Several socialization theories support the effect of parental behaviors and specifically of adaptive parenting strategies in determining the children’s attitudes to build positive social relationships compared to punishment strategies [28,29]. In particular, positive parenting was found to be positively related to children’s prosocial behaviors. In this term, previous studies conducted, for example, by Eisenberg et al. [30], showed that parents showing warmth, protection, and empathic concern toward their children, elicit in them a feeling of security and connectedness to others that is an important precursor of prosocial behaviors. Other studies highlighted that higher parental use of reasoning and explanation of behaviors (i.e., an example of supportive positive parenting) is associated with higher children’s prosocial behaviors [31,32]. In addition, positive parenting was associated with high parental expression of positive emotions in the interactions with their children which, in turn, was associated with encouraging their children’s expression of positive emotions and empathy in social relationships [33]. Furthermore, Serna and Martinez [24], in their study conducted on a sample of Spanish adolescents, showed that high parental involvement positively influences high adolescents’ prosocial behavior. In addition, other relevant contributions also support the role of other types of parenting styles in affecting adolescents’ social (mal)adjustment. We refer to the study conducted by Di Maggio and Zappulla [34] on Italian adolescents which showed that the authoritative, authoritarian, and indulgent styles are the most favorable for adolescents’ positive adjustment, and to the study conducted by Valente and colleagues [35] which demonstrated on a large sample of Brazilian adolescents that negligent parenting is the most associated with maladjustment.

To sum up, considering previous studies on the positive association between positive/authoritative parenting and youth prosocial behavior, it might be possible to hypothesize a positive association between supportive PES and youth prosocial behavior, as well as a negative association between unsupportive PES and youth prosocial behavior. Moreover, few studies investigated the role of culture in the associations between parenting and prosocial behavior. For example, Pastorelli et al. [26] found cross-cultural similarities in the association between positive parenting and youth prosocial behaviors. As already reported in the previous section, more studies are in need to explore, and eventually confirm, the direction of the aforementioned results from a cross-cultural perspective.

### 1.4. Parents’ Behaviors and Adolescents’ Emotion (Dys)regulation

Emotion regulation involves initiating, maintaining, and modulating emotional experience, intensity, and expression [36]. Empirical findings support the impact of family context and, more specifically, of parents’ behaviors in affecting youth’s emotion regulation capabilities [10,37]. In this term, Morris and colleagues [9] developed the tripartite model of the impact of the family on children’s emotion regulation and adjustment which supports the effect of parental behaviors in predicting youths’ emotional (dys)regulation. The tripartite model proposed by Morris includes three main processes, which, in a bidirectional influence, contribute to youths’ emotional socialization and regulation: (a) observation/modeling; (b) parenting practices; (c) family emotional climate [9]. Specifically, previous studies supported that an authoritarian parenting style was associated with less adaptive children’s emotional regulative strategies (e.g., [9,38]). It was hypothesized that those parenting practices could stimulate the children’s suppression of negative emotion and predict poor emotion regulatory skills [39]. Recently, Shaw and Starr [40], supported that parental emotion dysregulation in combination with an authoritarian parenting style increases conflicts in parent-child interactions and high children’s emotion dysregulation [41].

In contrast, an authoritative parenting style is associated with adaptive adolescents’ emotion regulation strategies by increasing support and compliance in the parent-child interaction [42,43]. Moreover, there are also previous studies in which it specifically emerged the association between supportive parental socialization strategies in response to children’s negative emotions and children’s emotion regulation (e.g., [44]).

In addition, cross-cultural similarities emerged in the association between harsh parenting and some youth emotion regulation-related indicators, such as either youth irritability, or internalizing and externalizing problems (e.g., [45]).

To sum up, even if the influence of parents’ behavior on youths’ emotion regulation is well established in the literature (e.g., [9]), several studies support that emotion regulation and dysregulation are influenced by cultural values, beliefs, and display rules associated with emotion (e.g., [46]). Therefore, more cross-cultural studies are in need to examine the impact of parents’ behaviors, and of PES in particular, on youth emotion (dys)regulation.

### 1.5. Parental Emotion Socialization in Cross-Cultural Perspective

When studying parental emotion socialization it is important to keep in mind the valence of the emotion being socialized by parents and the cultural contexts in which the families are embedded. The existing literature mainly focuses on parental socialization of children and adolescents’ negative emotions in North American samples (e.g., [9]). Recently, some researchers examined parental socialization in response to their children’s negative emotional reactions with a cross-cultural perspective. For example, Yeo et al. [8] compared three cultural contexts of a rural village in India, suburbs of Beijing, China, and an urban city in Singapore. Similarly, across the various cultures, it was found that unsupportive parental emotion socialization (e.g., punishment, neglect) was positively associated with adolescent internalizing and externalizing problems, whereas supportive parental emotion socialization (e.g., override and reward) was negatively associated with those behavioral problems. More studies are needed to understand whether parental emotion socialization similarly affects youth adjustment cross-culturally. This goal seems even more challenging when taking into account those studies attesting to cultural differences in the association between negative and positive emotions. Whereas a strong negative correlation between positive and negative emotions emerged in Western samples, whether a weak, not significant, or positive association emerged among some Asian cultural contexts [47]. In line with those results, it may be that parents from different cultural contexts may respond differently to their children’s emotional reactions, depending on the valence of the emotion expressed by their children. Thus, even though previous sections mainly showed cross-cultural similarities in the association between parenting and specific youth outcomes, such as school performance, prosocial behavior, and emotion (dys)regulation, the aim to explore the association between parental happiness socialization strategies and each of the aforementioned youth outcomes cross-culturally is exploratory.

### 1.6. Family Context in Italy and Azerbaijan

The purpose of this study is to compare the associations between PES and youth adjustment. To better contextualize this goal, little information is reported to describe overall how a family is considered in each of the examined cultural contexts. The family plays a key role in the life of Italians and has changed a lot in the last sixty years. In the past, Italian families were characterized by structured rigid roles and rules in the division of tasks between mothers and fathers compared to more recent years: mothers had the highest commitment to their child-care, while fathers generally took care of the economic maintenance of the family (e.g., [48]). However, after the ’50s, with the economic growth and industrialization that involved Italy, habits, and roles within the family context and parenting styles changed a lot (e.g., [48,49]). Regarding parenting and the gender role within the families, fathers have a less hierarchical role and even if mothers are predominantly involved in tasks related to the growth of their children, they also play an important role in the financial support of the family, and they are engaged in the world of work. In addition, Italian families are characterized by high levels of connectedness among the members and are warm and supportive towards adulthood [50]. Azerbaijan is a patriarchal society, meaning that fathers have a predominant role within the family; however, also mothers have an important role in what concerns the family [51]. Azerbaijani families are distinguished by their commitment towards high moral values, such as family, motherhood, and fatherhood [51]. In Azerbaijan, the value of motherhood is determined by qualities such as caring, devotion to children, attachment to children, feelings of love and compassion, loyalty to husband, and honesty. Furthermore, in Azerbaijan, people get married and start a family earlier than in many other societies.

### 1.7. The Present Study

The overall aim of the present study was twofold: (1) examining the potentially significant differences in the mean levels of parental happiness socialization strategies between Italy and Azerbaijan; (2) examining the predictive effects of parental happiness socialization in determining youths’ academic performance, prosocial behavior, negative emotions’ regulation, and dysregulation, while controlling for parents’ and adolescents’ gender and age, parents’ educational level, social desirability, and Covid-related problems. Regarding the first objective, no previous studies have investigated the cross-cultural differences between Azerbaijanis and Italian families; however, in accordance with the aforementioned literature [48, 49] bout the recent changes of Italian parenting styles which actually are less rigid compared to the past, and considering the history of Azerbaijan, it was expected to find higher unsupportive parental happiness strategies in Azerbaijan and higher supportive strategies in Italy regarding the second objective, in line with the literature previously described in the introduction, and in line with the tripartite model of the impact of parental behaviors on children’s emotion regulation and adjustment [9], it was hypothesized that supportive parental happiness socialization strategies would be positively associated with school performance, prosocial behaviors, and emotion regulation; whereas unsupportive parental happiness socialization strategies would be negatively associated with school performance, prosocial behaviors, and emotion regulation, and positively associated with emotion dysregulation.

Finally, our goal to examine the association between parental happiness socialization strategies and child adjustment in Italy and Azerbaijan is exploratory. However, on the base of studies conducted in different cultures [9, 47] we expect to find significant cultural differences in the impact of parental socialization strategies on the considered outcomes.

## 2. Materials and Methods

### 2.1. Participants

This study involved a cross-cultural sample of 846 parents (*M*_age_ = 45.11, *SD* = 8.29; 76.4% mothers) of Italians (N = 606, 81.9% mothers) and Azerbaijanis (N = 227, 61.4% mothers) youths (*M*_age_ = 12.89, *SD* = 4.06; 51% girls), collected during the COVID-19 pandemic; 71.63% of the total sample was recruited in Italy and 28.37% of the total sample in Azerbaijan. The years of education were 12.69 (*SD* = 4.43) for mothers from Italy, and 9.09 (*SD* = 5.91) for mothers from Azerbaijan, while the years of education were 12.02 (*SD* = 4.41) for fathers from Italy, and 10.07 (*SD* = 4.13) for fathers from Azerbaijan. In the sample, 74.9% of Italian mothers, and 59.8% of Azerbaijanis mothers reported working; among them, 69.8% of Italian mothers, and 58.9% of Azerbaijanis mothers worked full-time. The majority of the Italian families had an average gross annual income between 30,000 and 40,000 euros per year, while the majority of the Azerbaijanis families had an average gross annual income between 2.8500 Azn and 17,000 Azn.

### 2.2. Procedure

Data from Italian parents were collected during August and September 2020, December 2020, January 2021, and during March 2021, while data from Azerbaijanis parents were collected during June 2021. Parents from both cultures reported their opinion about their own and their youths’ behaviors during the COVID-19 pandemic.

After obtaining the approbation from the local Institutional Review Board, the informed consent information about the study’s objectives and the procedure was advertised through word of mouth, social media, and schools, and a URL link containing the survey in a set of questionnaires was created via the Qualtrics platform. The inclusion criteria to select participants were to be a parent of a child aged between 6 and 18 years old. After obtaining the consent, parents were invited to fill out the Qualtrics survey anonymously. Measures were administered in the dominant language of participants (Italian for Italian parents and Azerbaijanis for Azerbaijanis parents). Parents took about 30 minutes to fill in the survey. Due to the convenient nature of the sample, it was not possible to calculate the response rate. The average completion rate of the whole survey (including more measures than the ones included in this study) from both Italian and Azerbaijanis parents was 49.10%.

### 2.3. Measures

Socio-demographic variables. Parents’ gender (1 = mothers, 2 = fathers), youths’ gender, (1 = boys, 2 = girls) country (0 = Azerbaijan, 1 = Italy), and parents’ educational level (mothers’ and fathers’ years of education completed), were measured.

Covid-related problems. Parents completed 3 items (items: “Did you test positive for COVID-19?”, “Did your target child test positive for COVID-19?”, “Is there any person close to YOU and your family who has lost their lives due to the COVID-19 virus infection?”; Italy α = 0.75, Azerbaijan α = 0.71) to assess if they had or not (1 = no; 2 = yes) specific problems strictly related to the pandemic.

Parental social desirability. Italian parents completed the Lie Scale from the Big Five Questionnaire [52], which evaluates the tendency of participants to give a more positive image of themselves. Italian parents were asked to rate 11 items (item example: “I have never criticized anyone”) how typical each answer was for him/her (from 1 = “absolutely false for me” to 5 = “absolutely true for me”; α = 0.82). Azerbaijanis’ parents completed the Social desirability sale (Reynolds, 1982) and were asked if each of the items (e.g., “I’m always willing to admit it when I make a mistake”; α = 0.59) described them (1 = “Yes”) or did not describe them (0 = “No”). After computing the *Z* standardized scores (*M* = 0, *SD* = 1) for parental social desirability separately by culture, data from Italy and Azerbaijan were combined to have a total score of parental social desirability.

Parental socialization strategies in response to youths’ happiness (i.e., parental happiness socialization strategies). Parents completed on a 5-point Likert scale (from 1, “Never, almost never”, to 5, “Always, almost always”) 15 items from a new scale adapted by the parent’s version of the Emotion as a Child Scale, EAC [53,54], referred to the parental responses to the expression of their children’s happiness. Preliminary exploratory factorial analyses (EFA) showed the presence of two main dimensions of PES strategies (Further details about the EFA are reported in the Supplemental Materials): supportive strategies (6 items, e.g., When your child has been happy, how often did you help your son/daughter to appreciate the reasons that made him/her happy?”; Italy α = 0.81, Azerbaijan α = 0.80) and unsupportive strategies (eight items, e.g., When your child has been happy, how often did you tell your son/daughter that you do not approve his/her being happy?”; Italy α = 0.78, Azerbaijan α = 0.72) for the socialization of children’s happiness. An item (“When your child has been happy, how often did buy to your son/daughter something that he/she liked?”) was deleted because it was similarly and moderately associated with both factors [55]. Furthermore, the invariance function of these two sub-scales between Italy and Azerbaijan has been examined. The configurable model showed an acceptable fit, χ^2^ (39) = 175.66, *p* < 0.001, CFI = 0.96, RMSEA = 0.09 (90%CI 0.07, 0.10), SRMR = 0.05. Partial metric invariance was supported (Δχ^2^ (11) = 19.29, *p* = 0.06) by constraining all the factor loadings except for item six (supportive parental happiness socialization: “Did you ask your son/daughter what made him/her happy”) and 13 “(unsupportive parental happiness socialization: Did you get nervous when your son/daughter was happy?”) that were not invariant, χ^2^ (50) = 194.95, *p* < 0.001, CFI = 0.95, RMSEA = 0.08 (90%CI 0.07, 0.09), SRMR = 0.06. Finally, we retained partial scalar invariance (Δχ^2^ (11) = 19.29, *p* = 0.06) by further constraining all the intercepts except for item 2 (supportive parental happiness socialization: “Did you tell your son/daughter to contain his/her happiness?”), χ^2^ (62) = 215.25, *p* < 0.001, CFI = 0.95, RMSEA = 0.08 (90%CI 0.07, 0.10), SRMR = 0.07. Overall, since only three items out of fourteen showed a lack of invariance [56], we could assume that the constructs of supportive and unsupportive parental happiness socialization were measured in a similar way across Italy and Azerbaijan.

Youth academic performance. Parents completed on a 5-point Likert scale (from 1, “far below average”, to 5, “far above average”), their child’s academic performance in seven subject matters (reading, writing, grammar, mathematics, social studies, science, other subjects; Italy α = 0.87; Azerbaijan α = 0.86) from the Child Behavior Checklist (CBCL; [57]).

Youth prosocial behavior. Parents rated their child’s prosocial behavior on a 5-point Likert scale (from 1, “Never”, to 5, “Always”), three items (item example: “(S)he tries to help others”; Italy α = 0.83; Azerbaijan α = 0.85) from the scale of Caprara & Pastorelli [58], referred to the parents’ evaluation of their children’s degrees of helpfulness, sharing, and consoling.

Youth negative emotion (dys)regulation. Parents reported on a 5-point Likert scale (from 0, “Not at all”, to 5, “a lot”) their child’s negative emotion regulation and dysregulation on a modified version of the Difficulties in Emotion Regulation Scale (DERS; [58]) adjusted for three specific children’s negative emotions: anger, sadness, and anxiety. In particular, from the 36 items of the original version of the scale, ten items that better represented the construct of emotion (dys)regulation (with the highest factorial loadings per sub-scale reported in [58]) were selected and adjusted for anger, sadness, and anxiety [59]. For this study, 12 items were included for negative emotions’ adaptive regulation (e.g., “How often your child:—Even if he/she is angry/sad/anxious, he/she can control himself/herself.”; Italy α = 0.86, Azerbaijan α = 0.84), and 15 items for the negative emotions’ dysregulation (e.g., “How often your child:—Because he/she is angry/sad/anxious, it takes a long time for him/her Italy α = 0.91, Azerbaijan α = 0.90).

### 2.4. Data Analysis

Preliminary descriptive statistics (means, standard deviations; skewness, and kurtosis) of the variable of interest were investigated. In particular, the T-test comparison for an independent sample has been implemented to examine the potentially significant differences between Italian and Azerbaijani parents in the mean levels of parental happiness socialization strategies and of the other studied variables. Furthermore, Pearson’s correlation analyses have been implemented in order to preliminary investigate the potential significant associations among the studied variables for the total sample and separately for the country. Then, a multiple group path analysis via MPlus version 8 [60] was implemented to examine the differences between Italian and Azerbaijanis parents for what concerns the predictive effects of unsupportive and supportive PES strategies in response to child’s happiness reactions in affecting youths’ academic performance, prosocial behavior, negative emotions’ regulation, and negative emotions’ dysregulation. We considered parents’ and youths’ gender, youth’s age, parents’ educational level, parents’ social desirability, and Covid-related problems as covariates of all the examined variables. We also estimated the within-time correlations among the variables. We examined the potentially significant differences between cultures by estimating an unconstrained model where no parameters estimated were constrained to be equal across groups and we compared this model to a model where all structural paths were constrained to be equal across groups. If the Δχ^2^ between the constrained and unconstrained multigroup models was not significant (*p* > 0.05), meant that the constrained model of equality between groups was the best one. For each model, the following parameters were used to evaluate the model’s goodness of fit: Chi-square Goodness of Fit (χ^2^) with its degrees of freedom (*df),* Comparative Fit Index (CFI), Tucker-Lewis Index (TLI), Root-Mean-square Error of Approximation (RMSEA), Standardized Root-Mean-square Residual (SRMR). In addition to nonsignificant χ^2^, we also considered CFI and TLI values >0.90 [61,62], RMSEA < 0.07, and SRMR < 0.08 [61] as indicators of acceptable model fit.

## 3. Results

### 3.1. Descriptive Statistics and Mean Differences

Table 1 shows means, standard deviations, skewness, and kurtosis for the studied variables and the mean differences of the studied variables in relation to participants’ countries. All the variables were distributed at acceptable rates to evaluate their univariate normality (values less than 2 for univariate skewness and less than 5 for univariate kurtosis were used as criteria) [63].

Regarding the t-test for the independent sample, it emerged: higher supportive and unsupportive parental strategies for the socialization of children’s happiness, and higher negative emotions’ dysregulation and Covid-related problems in Azerbaijan compared to Italy; higher prosocial behavior and negative emotions’ regulation in Italy compared to Azerbaijan.

### 3.2. Correlation Analyses

Table 2 shows the correlations among the study variables, separately by country.

It emerged that supportive parental happiness socialization strategies were positively and significantly related with youths’ academic performance and prosocial behavior only in Aizerbajan; unsupportive parental happiness socialization strategies were negatively related with youths’ academic performance both in Italy and Aizerbajan and negatively and significantly related with youths’ negative emotions’ regulation only in Italy and with youths’ negative emotion dysregulation and Covid-reated problems only in Aizerbajan; youths’ academic performance was positively and significantly relted with youths’ prosocial behavior both in Italy and Aizerbajn, and negatively related with youts’ negative emotions’ dysregulation only in Italy and positively related with Covid-related problems only in Aizerbajan; youths’ prosocial behavior was negatively related with youths’ negative emotion regulation in Italy and positively related with this latter and with negative emotion dysregulation in Aizerbajan; youths’ negative emotion regulation were positively related with negative emotions’ dysregulation only in Aizerbajan and positively related to parents’ social desirability only in Italy; lastly, parents’ social desirability was negatively related with youths’ negative emotion dysregulation only in Aizerbajan.

### 3.3. Multi-Group Path Analysis

**Figure 1:**
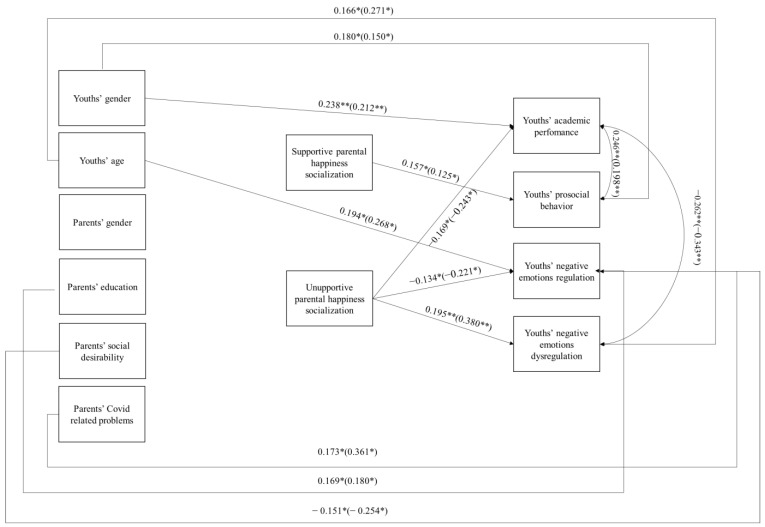
Multiple group path analysis model. Note. * = *p* < 0.05; ** = *p* < 0.01. Only significant paths are reported. Standardized coefficients in the constrained model (i.e., all coefficients are not significantly different across the two examined cultures) are presented separately for Italy and Azerbaijan (in parentheses).

We ran a multi-group path analysis model in which we examined the predictive effects of unsupportive and supportive PES strategies in response to child’s happiness reactions in affecting youths’ academic performance, prosocial behavior, negative emotions’ regulation, and dysregulation while considering parents’ and youths’ gender, youths’ age, parents’ educational level, parents’ social desirability, and Covid-related problems as covariates, and the within-time correlation. The path analysis cultures constrained model (Figure 1), in which the parameters were constrained to equality across Italy and Azerbaijan, reported a good fit to the data [χ^2^(56) = 60.61, *p* = 0.31, CFI = 0.95, TLI = 0.90, RMSEA = 0.03 (90% CI 0.00–0.08), SRMR = 0.10], and was not statistically different [Δχ^2^ (46) =53.65, *p* = 0.20] from the unconstrained model with freely estimated parameters [χ^2^ (10) = 6.96, *p* = 0.73, CFI = 1.00, TLI = 1.00, RMSEA = 0.00 (90% CI 0.00–0.10), SRMR = 0.03]. It emerged that: (a) supportive PES happiness strategies were positively associated with youths’ prosocial behavior; (b) unsupportive PES happiness strategies were negatively associated with youths’ academic performance and negative emotion regulation and positively related with youths’ negative emotions’ dysregulation. We found a positive within-time correlation between youths’ academic performance and prosocial behavior, and a negative within-time correlation between youths’ academic performance and negative emotions’ dysregulation. Furthermore, it emerged that being a girl was associated with higher academic performance and prosocial behavior and that higher parents’ educational level was associated with higher youths’ prosocial behavior. Furthermore, youths’ age was positively associated both with higher negative emotion regulation and dysregulation. Furthermore, a higher social desirability was associated with higher negative emotion regulation. In addition, it emerged that parents’ Covid-related problems were associated with lower youths’ negative emotional regulation.

## 4. Discussion

Theoretical and empirical findings highlight the relevance of socialization within the family context and of parents’ behaviors towards children’s emotional reactions, in affecting youths’ (mal)adjustment (e.g., [10]). In this term, recent studies (e.g., [6]) supported the key role played by parental socialization for negative emotions on youth adjustment, even if, there is a lack of studies focused also on the socialization of positive emotions (e.g., happiness). Accordingly, the present study aims to examine the impact of nowadays parents’ reactions to their children’s happiness and youths’ socio-emotional adjustment, at cross-cultural level. Specifically, this study has two main objectives: (1) to examine the mean differences in the parental socialization strategies for happiness and youth adjustment between Italian and Azerbaijan; (2) to examine the association between parental happiness socialization and youth adjustment. The following indicators of youth adjustment were examined: academic performance, prosocial behavior, negative emotions’ regulation, and dysregulation. Considering the data examined in this study were collected during the unique historical event that affected all people in the world and that has dramatically changed individuals’ habits and lifestyles at the cross-cultural level, which is the COVID-19 pandemic, the aforementioned aims were examined controlling for a set of covariates (e.g., child age and gender, parental education, and social desirability), including COVID-19-related problems. Regarding the first objective, as expected the t-test comparison for the independent sample showed higher unsupportive parental strategies for the socialization of children’s happiness in Azerbaijan in comparison to Italy; however, in contrast with our expectations, the results also showed higher supportive parental strategies in Azerbaijan compared to Italy. This unexpected result may be explained by the fact that, even if Azerbaijan is a patriarchal society, characterized by rigid roles and tasks, Azerbaijani mothers are fully and primarily engaged in their children’s care and growth.

Regarding the second objective, based on Pearson’s correlations, the study’s findings supported the presence of significant associations among the variables. In particular, consistent with previous studies which suggest the impact of positive parenting on youths’ academic performance (e.g., [64]), prosocial behaviors (e.g., [26]), and emotion regulation, and positive association between negative parenting and youths’ emotion dysregulation (e.g., [37,65]), it emerged that supportive parental happiness socialization strategies were positively related with youths’ academic performance and prosocial behavior. In addition, findings supported negative associations between unsupportive parental strategies for the socialization of children’s happiness and academic achievement, prosocial behaviors, and emotional regulation, and positive associations with emotion dysregulation. These results confirm the significant associations between supportive parental happiness socialization strategies and youths’ adjustment and between unsupportive parental happiness socialization strategies and youths’ maladjustment. Finally, based on the multiple-group path analysis, the predictive effects of both supportive and unsupportive parental socialization strategies on youths’ (mal)adjustment, while controlling for the effects of youths and parents’ gender, youths’ age, parental educational levels, parents’ social desirability, and Covid-related problems, were similarly confirmed both in Italy and in Azerbaijan. As expected, supportive parental happiness socialization strategies positively and significantly predicted youths’ prosocial behavior. However, contrary to our expectations, supportive parental happiness strategies did not predict academic achievement, which was negatively predicted by unsupportive parental happiness socialization strategies, and they did not predict youths’ negative emotion regulation and dysregulation. Furthermore, unsupportive parental happiness socialization strategies positively and significantly predicted youths’ negative emotional dysregulation and negatively predicted youths’ negative emotion regulation. Furthermore, according to recent studies, (e.g., [6]) which showed the impact of the COVID-19 pandemic on youths’ adjustment, results showed that Covid-related problems were negatively associated with youths’ negative emotion regulation. The results showed no significant differences in all the examined associations between Italy and Azerbaijan. Overall, our findings are in accordance with the tripartite model of the impact of parenting on children’s emotion regulation and adjustment [9]; however, our results also suggest different impacts of supportive and unsupportive parental socialization practices, showing that supportive parental strategies seem to act only on the social adjustment, while unsupportive strategies seem contrasts the youths’ emotion regulation capabilities and scholastic achievement. These results provide new insights into the role of specific parenting behaviors that deserve to be further investigated, also considering the impact of the stressful event that affected families during data collection, and the whole world, i.e., the COVID-19 pandemic.

### Strengths, Limitations, and Future Directions

This study presents several strengths. First, it increases theoretical and empirical knowledge about parental socialization for youths’ happiness and about their impact on youths’ academic and socio-emotional adjustment in the framework of the COVID-19 pandemic and at the cross-cultural level. Second, significant results regarding the path analyses model emerged while controlling for parental social desirability, educational level, youths, and parents’ gender, youths’ age, and parents’ Covid-related problems also, considering at the same time a sample composed of both mothers and fathers, that allows greater generalizability of the study’s findings. Lastly, this study includes a cross-cultural sample that allows some generalizability of the findings across different cultural contexts.

This study has also some limits. First, we used two convenient samples that cannot be considered nationally representative of the general population. Second, we did not examine the role played by other variables such as youths’ temperament, parents’ personality traits, youths’ behavioral problems (internalizing and externalizing symptoms), and parents’ socialization toward their children’s negative emotions. Another relevant limit of the present study consists of the relatively low participation rate. We can speculate that this issue could depend on the strategy used to recruit participants which was principally based on the spread of the link containing the survey without having direct contact with potential participants that could be useful for providing clearer explanations about the study. Furthermore, another important limit related to the strategy used to recruit participants consists in the imbalance of the participation rate between Italy and Azerbaijan and between mothers and fathers. A further limitation of this study consists in the low alpha for the Social Desirability scale [66]; future studies should further examine the source of validity problems in this scale. Another important limitation of the study consists in the fact that the measures we used were only parent-reported; in fact, adolescent reports could help to better identify the specific type and effect of parental happiness socialization strategies on their adjustment and emotional regulation. Furthermore, we cannot exclude that the consideration of youths’, and not only parents’ perceptions could produce different results. Future research should rely on a multi-informant approach based also on child-reported measures. A further limitation of this study consists in the label used for the constructs of parental emotion socialization strategies, which could seem too general; future studies should more in detail examine their adaptive and maladaptive effects on youths’ adjustment.

Lastly, our data are correlational in their nature limiting causal relationships among the examined variables.

Future studies should overcome these limitations by examining parental socialization strategies in relation to a wide range of discrete emotions (both positive and negative), investigating other individuals’ outcomes, and considering other cultures. Finally, in accordance with the theoretical framework of bidirectional reciprocal exchanges between parents and children [67], an alternative model could be tested in addition to the one examined in the present study, in which youths’ adjustment predicts parental emotion socialization.

## 5. Conclusions

The present study has several implications for parenting in the 21st Century, particularly taking into account that this study is embedded in the peculiar adverse worldwide adverse context of the COVID-19 pandemic. In particular, the study’s results contribute to the understanding of the association between parental behaviors in response to their children’s reactions to happiness and youth (mal)adjustment. Those results emerged taking into account the cultural contexts in which the families were embedded, and no significant results emerged on a cross-cultural level. That can be interpreted as proof of the universal power of parental emotion socialization in impacting youth socio-emotional development. In addition, those results emerged while controlling for many covariates, including Covid-related problems. Specifically, it did emerge a more disadvantageous scenario, in terms of worst socio-emotional adjustment, for those youths whose families had to deal with many problems related to the COVID-19 pandemic (e.g., having tested positive for COVID-19 or having lost someone because of it) than those whose families had fewer problems related to Covid.

These study’s findings provide some useful directions in terms of possible actions professionals may take to promote youth emotional healthy development and positive interpersonal relationships (i.e., positive youth development; [68]), through encouraging parental use of supportive, rather than unsupportive, strategies in response to their children’s positive emotional reactions. This aspect could promote positive parenting behaviors capable of predicting the adaptive development of their children, at the net of the parenting challenges and complex tasks that 21st-century society requires of parents.

Following this direction, the study findings could be translated in terms of intervention programs aimed at increasing the use of what are defined as supportive practices of socialization of children’s emotions with implications also in terms of public policies aimed at helping families who experience stressful life events in socializing their children. In these terms, future studies aimed at investigating the effect of specific parental behaviors on the socialization of children’s emotions also in other cultural contexts would be useful, which could therefore allow a wider generalization of the results and the international application of intervention policies.

## Figures and Tables

**Table 1:** Descriptive statistics and mean differences.

		Mean	SD	Skewness	Kurtosis	T-Test for Independent Samples
Supportive parental happiness socialization	Italy	4.05	0.72	−0.80	0.71	3.68 **^a^
	Azerbaijan	4.26	0.71	−1.45	3.19	3.71 **^b^
Unsupportive parental happiness socialization	Italy	1.83	0.66	1.04	1.91	23.06 **^a^
	Azerbaijan	3.13	0.85	−0.40	−0.75	20.59 **^b^
Youth academic performance	Italy	3.26	0.54	−0.64	−0.00	0.35 ^a^
	Azerbaijan	3.28	0.60	−1.17	2.05	0.36 ^b^
Youth prosocial behavior	Italy	3.66	0.83	−0.26	−0.23	−5.65 **^a^
	Azerbaijan	2.95	1.08	0.08	−0.69	−6.01 **^b^
Youth negative emotions regulation	Italy	2.97	0.71	−0.54	0.13	−4.17 **^a^
	Azerbaijan	2.57	0.74	0.12	−0.25	−4.19 **^b^
Youth negative emotions dysregulation	Italy	2.07	0.66	0.58	−0.15	3.94 **^a^
	Azerbaijan	2.45	0.79	0.46	−0.74	4.04 **^b^
Parents’ social desirability	Italy	−0.00	1.00	−0.28	−0.55	0.08 ^a^
	Azerbaijan	−0.01	0.77	0.24	−0.55	0.08 ^b^
Covid-related problems	Italy	1.12	0.21	1.75	2.77	15.91 **^a^
	Azerbaijan	1.69	0.33	−0.77	−0.56	17.97 **^b^

Note. *SD* = Standard deviation. ** = *p* < 0.01; * = *p* < 0.05. ^a^ = equal variances assumed T test statistic; ^b^ = equal variances not assumed T test statistic.

**Table 2:** Correlation analyses separately for country.

	(1)	(2)	(3)	(4)	(5)	(6)	(7)	(8)
(1) Supportive parental happiness socialization	- ^a^							
	- ^b^							
(2) Unsupportive parental happiness socialization	−0.002 ^a^	- ^a^						
	0.035 ^b^	- ^b^						
(3) Youth academic performance	0.024 ^a^	−0.228 **^a^	- ^a^					
	0. 334 **^b^	−0.187 **^b^	- ^b^					
(4) Youth prosocial behavior	0.140 ^a^	−0.141 ^a^	0.284 **^a^	- ^a^				
	0. 333 **^b^	−0.103 ^b^	0.186 *^b^	- ^b^				
(5) Youth negative emotion regulation	0.155 ^a^	−0.330 **^a^	0.075 ^a^	−0.254 *^a^	- ^a^			
	0.093 ^b^	−0.031 ^b^	−0.074 ^b^	0.469 **^b^	- ^b^			
(6) Youth negative emotion dysregulation	0.019 ^a^	0.132 ^a^	−0.253 **^a^	−0.047 ^a^	−0.076 ^a^	- ^a^		
	0.028 ^b^	0.169 *^b^	−0.027 ^b^	0.304 **^b^	0.541 **^b^	- ^b^		
(7) Parents’ social desirability	0.046 ^a^	0.028 ^a^	0.025 ^a^	0.001 ^a^	0.264 **^a^	−0.005 ^a^	- ^a^	
	0.005 ^b^	−0.062 ^b^	0.063 ^b^	−0.019 ^b^	−0.109 ^b^	−0.296 **^b^	- ^b^	
(8) Covid-related problems	−0.122 ^a^	0.226 **^a^	0.022 ^a^	−0.017 ^a^	−0.122 ^a^	−0.020 ^a^	−0.009 ^a^	- ^a^
	0.066 ^b^	−0.146 *^b^	0.144 *^b^	−0.062 ^b^	−0.019 ^b^	−0.038 ^b^	0.065 ^b^	- ^b^

*Note*. ** = *p* < 0.01; * = *p* < 0.05. ^a^ = Italy; ^b^ = Aizerbajan.

## Data Availability

Data available on request due to restrictions, e.g., privacy or ethical. The data presented in this study are available on request from the corresponding author. The data are not publicly available due to the privacy and professional specificity of the people who took part in this research.

## Supplementary Materials

The following are available online at https://www.mdpi.com/article/10.3390/ijerph20043604/s1, Figure S1: Graph of the eigenvalues for the items of the socialization of happiness; Table S1: Pattern matrix for the two-factors solution for the items of the socialization of happiness.
