# Supplementary material for: Parental Happiness Socialization and Youth Adjustment in Italy and Azerbaijan in the COVID-19 Pandemic Era
Source: Int J Environ Res Public Health. 2023 Feb 17;20(4):3604. doi: 10.3390/ijerph20043604 (PMC9960549; doi:10.3390/ijerph20043604)
Supplement: Supplementary file 1 [file ijerph-20-03604-s001.zip › ijerph-2203836-supplementary.pdf]

Supplemental Materials

Exploratory Factorial Analyses (EFA) on the Parental Emotion Socialization Strategies in response to child' s happiness

Exploratory Factorial Analyses (EFA) were implemented by using SPSS.19 Statistical Software.

Figure S1 reported the scree plots from the EFA on the items of parental socialization strategies in response to children's happiness.

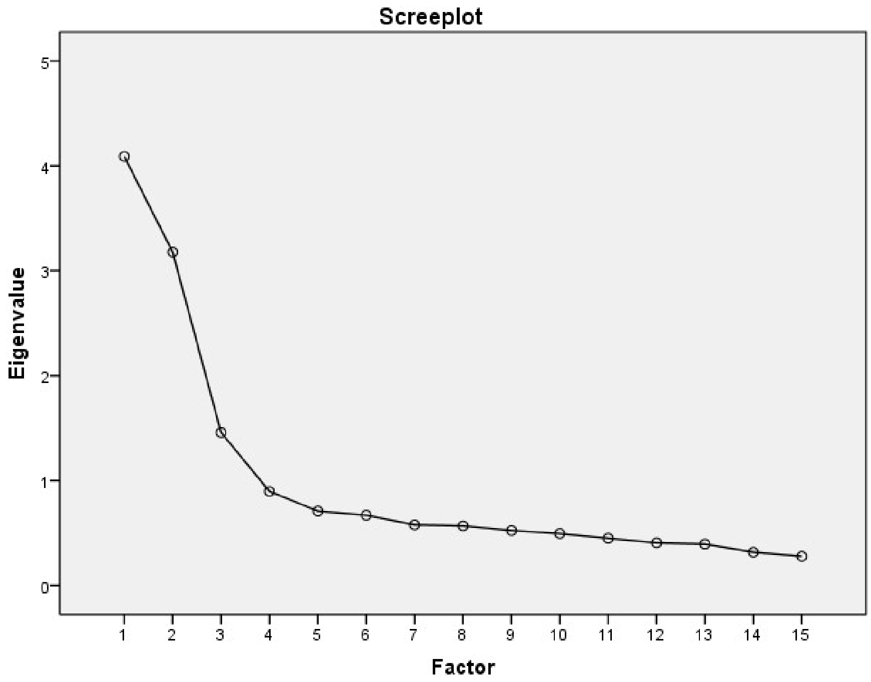

Figure S1. Graph of the eigenvalues for the items of the socialization of ***happiness***

In the graphs reported in Figure S1, a two-factor factorial solution seems somehow to emerge for parental socialization of happiness.

The pattern matrix of 5, 4, 3 and 2 factor solutions were also preliminarily investigated. But only the 2-factor solution provided a comprehensible solution in line with the original scale. Table S1 shows the matrix of the two-factor solution

Table S1. Pattern matrix for the two-factors solution for the items of the socialization of happiness

When your child has been HAPPY, how often….?	*Unsupportive*	*Supportive*
1.Did you point out to your son/daughter that you were aware of her/his happiness?	.077	**.708**
2. Did you tell to your son/daughter to contain his/her happiness?	**.680**	.290
3. Did you help your son/daughter to appreciate the reasons that made him/her happy?	.129	**.696**
4. Did you feel happy for your son/daughter's happiness?	-.057	**.567**
5. Did you tell to your son/ daughter that the way in which (s)he expressed his/her happiness made him/her looking like younger than his/her own age?	**.777**	.249
6. Did you ask to your son/daughter what made him/her happy?	.109	**.679**
7. Did you tell your son/daughter to take care of other things?	**.732**	.226
8. Did you show to your son/daughter that you feel happy for his/her happiness?	.045	**.712**
9. Did you tell your son/daughter that you do not approve his/her being happy?	**.637**	.055
10. Did you buy to your son/daughter something that he/she liked?	~~.410~~	~~.431~~
11. Did you tell your son/daughter to shut up when he/she was happy?	**.707**	-.011
12. Did you take some time to think about your son/daughter?	.125	**.511**
13. Did you get nervous when your son/daughter was happy?	**.317**	-.169
14. Did you not pay attention to your son/daughter happiness?	**.494**	-.041
15. Did you tell your son/daughter to keep calm when he/she was happy?	**.523**	-.009

Overall, it emerged one factor including the items originally described by Klimes-Dougan et al. (2007) as indicators of strategies typically considered supportive emotion socialization strategies, while a second factor refers to items related strategies typically considered unsupportive. We choose to eliminate item 10 ("Did you buy to your son/daughter something that he/she liked?"), as it saturated almost at the same rate on the two extracted factors.
